# Neuro-Immunity and Gut Dysbiosis Drive Parkinson’s Disease-Induced Pain

**DOI:** 10.3389/fimmu.2021.759679

**Published:** 2021-11-18

**Authors:** Katiane Roversi, Natalia Callai-Silva, Karine Roversi, May Griffith, Christos Boutopoulos, Rui Daniel Prediger, Sébastien Talbot

**Affiliations:** ^1^ Département de Pharmacologie et Physiologie, Université de Montréal, Montréal, QC, Canada; ^2^ Centre de Recherche Hôpital Maisonneuve-Rosemont, Montréal, QC, Canada; ^3^ Département d’Ophtalmologie, Université de Montréal, Montréal, QC, Canada; ^4^ Departamento de Farmacologia, Universidade Federal de Santa Catarina, Florianópolis, Brazil

**Keywords:** Parkinson’s disease, pain, nociceptor neurons, neuro-immunity, microbiota, dysbiosis, gut-brain axis

## Abstract

Parkinson’s disease (PD) is the second most common neurodegenerative disorder, affecting 1–2% of the population aged 65 and over. Additionally, non-motor symptoms such as pain and gastrointestinal dysregulation are also common in PD. These impairments might stem from a dysregulation within the gut-brain axis that alters immunity and the inflammatory state and subsequently drives neurodegeneration. There is increasing evidence linking gut dysbiosis to the severity of PD’s motor symptoms as well as to somatosensory hypersensitivities. Altogether, these interdependent features highlight the urgency of reviewing the links between the onset of PD’s non-motor symptoms and gut immunity and whether such interplays drive the progression of PD. This review will shed light on maladaptive neuro-immune crosstalk in the context of gut dysbiosis and will posit that such deleterious interplays lead to PD-induced pain hypersensitivity.

## Pain

Pain is defined as an unpleasant sensory and emotional experience associated with real or potential injury (1). Physiologically, pain serves as a protective mechanism, alerting the host to environmental danger. The sensation results from the integration of complex neurobiological systems that detect, integrate, and coordinate protective responses to noxious stimuli that threaten the host’s homeostasis and survival (2).

Nociceptors express various ion-channel receptors that are specialized to respond to threats posed by pathogens, allergens, and pollutants. Nociception is then initiated upon sensing these noxious stimuli by first-order neurons which, once activated by their cognate ligands, allow the influx of cations (Na^+^, Ca^2+^) leading to the generation of an action potential. These electrical signals are then propagated through the length of small, unmyelinated C or myelinated Aδ fibers to the spinal cord, where they synapse with second-order neurons (3). These electrical signals are then modulated—either amplified or blunted—by local immune cells or descending neurons.

The descending pathways originate from supraspinal structures such as the rostral ventromedial medulla (RVM), the dorsolateral pontomesencephalic tegmentum, and the periaqueductal gray matter (PAG). The descending pathways inhibit pain by releasing monoamines, such as dopamine, norepinephrine, and serotonin into the dorsal horn. Additionally, endogenous opioids exert descending inhibition of nociception (4).

Once modulated in the spinal cord, the nociceptive signal is processed in the supraspinal region and recognized as pain. The lateral pain system comprises the spinothalamic tract, which projects through the lateral thalamus and toward the sensory cortical areas, and is primarily involved in processing sensory discrimination, localization, and pain intensity. In contrast, the medial pain system processes the motivational-affective and cognitive-evaluative aspects of pain (e.g., unpleasantness, suffering) and projects through the medial thalamic nuclei toward the anterior cingulate cortex (5).

## Chronic Pain

Chronic pain affects approximately 20% of the general population and individual of any age. It negatively impacts the patient’s quality of life and is also associated with mood and sleep disorders. It is considered the chief debilitating symptom of a variety of diseases, ranging from cancer to multiple sclerosis (6–8). While acute pain serves a physiological purpose, chronic pain is rarely self-resolving and remains resistant to pharmacological treatment (9). This pain can be either perceived more severely, a phenomenon known as hyperalgesia, or it can be generated by non-noxious stimuli, a condition known as allodynia.

Chronic pain results from persistent and repeated stimuli which can lead to peripheral and/or central sensitization of nociceptor neurons. Specifically, central sensitization is characterized by the persistent hyperexcitability of the central nervous system (CNS) circuitry triggered by excessive neuronal activity resulting from peripheral tissue inflammation or neuropathic injury. Central sensitization can result from i) modifications of glutamatergic receptors; ii) the upregulation of proteins involved in maintaining synaptic strength; iii) Aβ neuron sprouting; iv) decreased inhibitory control by GABAergic interneurons; and v) increased expression of activator ion-channel or neuropeptide receptors (10).

Secondary to the actions of pro-inflammatory cytokines released by microglia and astrocytes, these modifications increase the synaptic activity between first- and second-order neurons by altering the biophysical properties of danger-detecting ion-channel receptors and promoting the trafficking of these receptors to the synaptic membrane. Functionally, the sensitization is reflected by a long-term potentiation of the synaptic transmission between primary and second-order sensory neurons, rendering the CNS hypersensitive to normal (or previously innocuous) inputs. Clinically, this higher level of excitability maintains chronic pain (10–12).

## Immunity Drives Pain

Along with detecting adverse temperature, pressure, and chemicals, nociceptor neurons express specific receptors for numerous immunoglobulins, cytokines, and chemokines. The nociceptor neurons are tuned to detect and respond to mediators derived from immunocytes (13–16). Typically, the binding of these sensitizing molecules generates intracellular signaling *via* tyrosine kinase or G-protein-coupled receptors (15, 17, 18). The second messengers downstream of these receptors trigger i) the phosphorylation and membrane expression of ion-channel receptors and voltage-gated sodium channels; and ii) the overproduction of neuropeptides and neurotransmitters (19–21).

A few well-known examples of immunocyte-releasing pain-sensitizing mediators include the action of interleukin 1 beta (IL-1β) (22–24), tumor necrosis factor (TNF-α) (25, 26), prostaglandin E2 (PGE_2_) (27), and nerve growth factor (NGF) (24). For example, PGE_2_ stimulates nociceptor-expressed DP2, which leads to an increase in protein kinase A (PKA) and protein kinase C (PKC) activity, which, in turn, phosphorylates transient receptor potential vanilloid 1 (TRPV1). Consequently, PGE_2_ increases capsaicin-induced currents, as found in cultured rat dorsal root ganglion (DRG) neurons (28, 29). In the case of NGF, when binding to its cognate receptor TrkA, it triggers PI3K/Src kinase activation which also leads to the phosphorylation of TRPV1 (18, 30).

These mechanisms stem from work done on models of rodents with nerve injury or auto-immune diseases. While no particular neuro-immune cascade appears to be the primary driver of the pain sensation, data show the involvement of central and peripheral innate (i.e. macrophages) and adaptive (i.e. T cells) immune cells. While typically studied in isolation, peripheral and central mechanisms should be studied concurrently. This is exemplified by the data showing that monocytes and microglia synergize in driving neuropathic pain in mice with nerve injuries. Thus, both types of cells need to be eliminated to alleviate pain. However, when used separately, monocyte depletion using clodronate liposomes or CX3CR1^+^ microglia ablation failed to impact the course of the disease (31).

In a model of chronic constriction injury (CCI), T cells are recruited to the sciatic nerve and induce mechanical allodynia and thermal hyperalgesia *via* the production of IL-17A and IFN-γ (32). Athymic nude male rats, which have no T cells, were protected. Upon nerve injury, T cells infiltrate the DRG and release leukocyte elastase (LE) which promotes mechanical allodynia (33–35) while spinal nerve transection-induced neuropathic pain was found to be mediated by T_H_1 cells released IFN-γ, TNF-α, and GM-CSF (36). Interestingly, when comparing the spinal dorsal horn gene profile of spared nerve injury (SNI) animals, T cells and glia seem predominately impacted in adult mice rather than younger animals. These data may indicate age-dependent neuromodulation by immune cells, which could explain why hypersensitivity seems to increase with age.

Neuro-immune crosstalks are not limited to the site of injury, as shown in models of chemotherapy and sciatic nerve ligation. Immunocytes (i.e., macrophages, monocytes, neutrophils, and T cells) infiltrate the DRG in a TLR2- or CCL-2-mediated fashion (37, 38). Once in the tissue, they release IL-1β and TNF-α, which can cause thermal hyperalgesia (39–44). In contrast, the targeted depletion of IL-10-producing monocytes and macrophages delayed pain resolution (45).

Aside from these traditional mechanisms, antibodies produced in auto-immune diseases can initiate pain. For instance, the injection of autoantibodies against citrullinated proteins (ACPA) purified from animals with rheumatoid arthritis promotes pain-like behavior without inflammation. This is achieved by acting on osteoclasts and inducing CXCL1, a human analog of IL-8 (46). In addition, IgG from patients with complex regional pain syndrome (CRPS) prolonged postsurgical hypersensitivity to mechanical, cold, and heat stimuli. Finally, skin-saphenous nerve preparations from tCRPS mice show increase sensitivity to auto-antibodies (47), which impairs the function of the potassium channel Kv1.2 and promotes mechanical hypersensitivity (48).

Another novel mechanism of pain modulation was described by Chen et al., who found that by binding to peripheral sensory neuron-expressed PD-1, the immune checkpoint ligand PD-L1 triggers the phosphorylation of SHP-1 and the downstream modulation of sodium and potassium channels. Consequently, PD-L1 suppressed excitatory synaptic transmission (sEPSC) in lamina II neurons of the spinal cord which trigger analgesia (49).

Beyond the commonly known Pattern recognition receptor (PRR) typically expressed by immune cells, Stimulator of interferon genes protein (STING1) was recently found to be abundant in TRPV1^+^ neurons (50, 51). Its activation by IFN-I ligands led to long-lasting analgesia by suppressing the excitability of nociceptors through the modulation of sodium and calcium channel function (51).

## Microbes Induce Pain

The Gastrointestinal (GI) tract is innervated by intrinsic neurons from the enteric nervous system (ENS) and by the axons of extrinsic sympathetic, parasympathetic, and visceral afferent neurons (52, 53). The ENS is organized into two major neuronal networks—the myenteric plexus, and the submucosal plexus—and also comprises connective interneurons and various types of supporting glial cells. The ENS sympathetic (noradrenergic) neurons control blood vessel vasocontraction, while the parasympathetic (cholinergic) neurons control gut contraction (54).

The gut’s extrinsic innervation is made up of neurons originating from lumbar (DRG) and nodose (ND) ganglia. These neurons work to monitor GI volume and intestinal contents, while the gut hormones regulate the digestive physiology (55). The DRG neurons project along the mesenteric arteries, while the ND ganglion neurons project from the vagus nerve.

The vagus, which consists of ~2,300 sensory neurons, projects to half of the large intestine. The GI tract innervation accounts for ~20% of its terminals (56). Most of these extrinsic fibers (DRG and ND) express sensory neuron markers such as TRP channels (TRPV1) (57), voltage-gated sodium channels (Na_V_1.8) (58), and mechanosensitive channels (Piezo2) (59). These sensory neurons are designed to limit tissue damage by detecting and initiating protective reflexes (60, 61).

Under homeostatic conditions, the lumen of the intestine is not directly innervated, meaning that there is no direct neuron sensing of the luminal content (50, 62). The signals are sent by intestinal enteroendocrine cells which expressed glutamate receptors and can release a few neuropeptides (i.e., cholecystokinin, peptide YY (PYY)), consequently enabling the perception of the luminal content by vagal neurons (63). Upon penetrating the epithelial barrier, as in the case of a lesion or an infection, the proteases, reactive oxygen species, or cytokines produced by mucosa-resident immune cells may stimulate the ENS neurons (64, 65).

Different making in gut pathogens, as found in dysbiosis, is associated with headaches, chemotherapy-induced neuropathic pain, and abdominal pain. *Staphylococcus aureus* heightens sensory hypersensitivity *via* membrane-bound N-formylated peptides or by releasing various pore-forming toxins (i.e., alpha-hemolysin, phenol-soluble modulins, leukocidin). Conversely, gut nociceptor-released Calcitonin gene-related peptide (CGRP) regulates M cell density, limiting pathogen entry into the GI tract (66). TRPV1^+^ neurons also appear to be associated with mucosal resistance against *Candida albicans*. They do so by increasing CD301b^+^ dDCs release of IL-23 and subsequent production of IL-17A from γδ T cells (67). Finally, the recognition of soluble bacterial products by ENS axonal termini, as found in the microfluidic gut model, drive RORλ^+^ T_reg_ induction and immunosuppression (68).

## PD-Induced Pain

Pain is a non-motor symptom present in 60–85% of PD patients (6, 69, 70). PD-induced pain negatively impacts a patient’s quality of life (8) and may exacerbate other non-motor PD symptoms such as depression and sleep disorders (7). In approximately one-third of PD sufferers, pain precedes the onset of PD motor symptoms by several years (71, 72). Peripheral neuropathic pain is also twice as frequent in PD patients (73, 74). Conversely, patients experiencing chronic pain are at increased risk of developing PD (75).

Pain often manifests as a musculoskeletal hypersensitivity affecting the neck, arms, or paravertebral muscles. It is believed to be a consequence of PD motor symptoms. PD can also trigger visceral pain, which affects the internal organs and results from the activation of nociceptors localized in the thoracic and pelvic organs. Visceral pain is associated with gastrointestinal dysfunction, as seen in PD patients (76). Finally, neuropathic pain is observed in 4%–10% of PD patients (77–79) and is typically associated with lesions in the central or peripheral nervous system (80). Clinically, it presents as burning, cramping, aching, numbness, tingling, vibrating, or lancinating sensations. This type of pain may be associated with autonomic manifestations, and it often stems from the face, head, pharynx, epigastrium, abdomen, pelvis, rectum, and genitalia. This type of pain does not correlate with the severity of motor impairments and often precedes their onset (79, 81–84).

Despite its clinical relevance and predictive value, pain is often neglected or misdiagnosed in PD patients and remains poorly managed (85). The contribution of maladaptive peripheral (*Peripheral Mechanisms of PD-Induced Pain*) and central (*Central Mechanisms of PD-Induced Pain*) neuro-immune interplays as well as gut-brain axis dysregulation (sections *GI Dysfunction in PD-Induced Pain* and *Dysbiosis in PD-Induced Pain*) (86) to the induction of PD-induced pain will now be discussed.

## Immunity in PD

Based on the overall role that neuro-immunity plays in sustaining sensory hypersensitivity (*Immunity Drives Pain*), we posit that neuronal loss or damage leads to the local recruitment of immune cells. It is well established that inflammation drives PD progression (87), involves innate and adaptive immune cells, and can occur in the peripheral or CNS. An indication that the immune system is responding to tissue damage stems from increased TNF-α, IL-1β, IL-2, IL-6, IFN-γ, and CCL2 levels observed in the blood and cerebrospinal fluid (CSF) of PD patients (88–91). These increases correlate with disease progression. For example, higher serum levels of TNF-α were linked to motor dysfunction, while raised levels of IL-1β and IL-2 were associated with cognitive decline (92).

**Figure 1:**
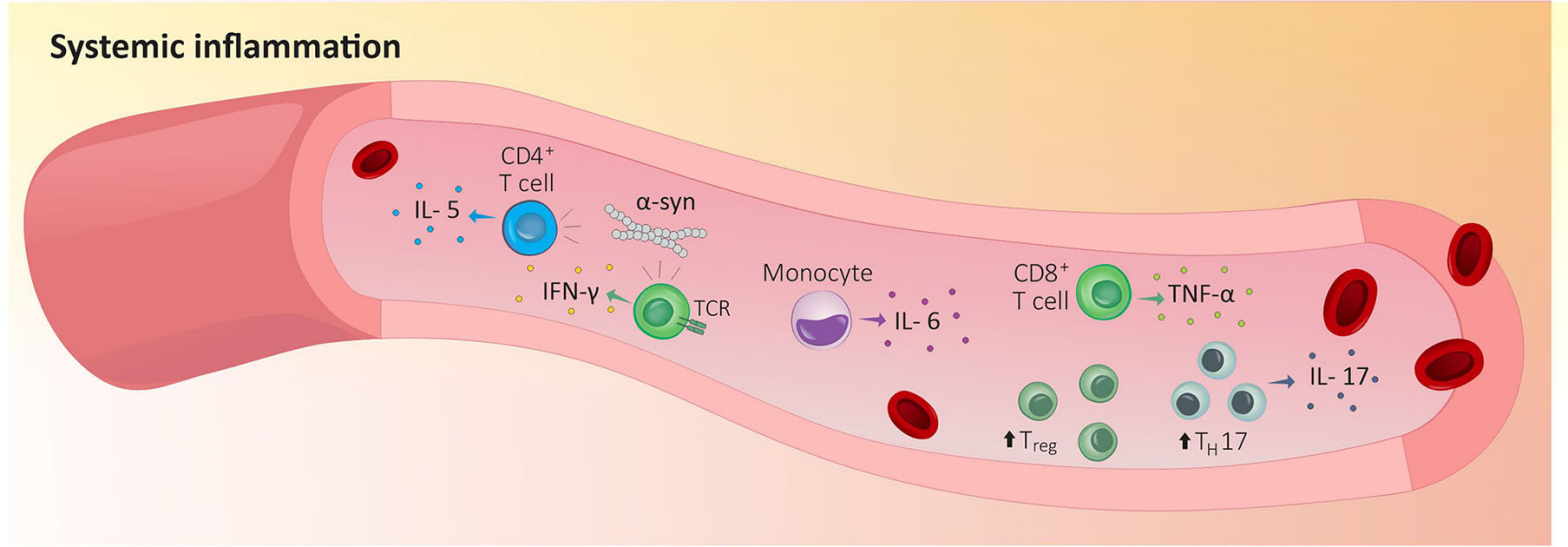
Peripheral inflammation in Parkinson’s disease. Indicators that the immune system is responding to tissue damage stems from increased blood-circulating T_reg_, activated CD4^+^ T cells, IL-17-producing T_H_17 cells and IL-6-producing monocytes in PD patients’ blood. In addition, circulating α-syn-reactive CD4 T cells are expanded in PD patients’ blood and blood purified CD4^+^ and CD8^+^ T cells from PD patients recognize and respond to α-syn which increase the production of IL-5 or IFN-γ. IFN-γ, interferon γ; IL-5, interleukin 5; IL-6, interleukin 6; IL-17, interleukin 17; T_H_17, T helper 17 cells; TCR, T cell receptor; TNF-α, tumor necrosis factor; T_reg_, regulatory T cells; α-syn, α-synuclein .

This increase in circulating cytokines was secondary to a rise in the number of immunocytes in a patient’s bloodstream. As expected, the severity of the disease correlates with lower levels of naïve CD4^+^ T cells (93–95) but increased levels of blood-circulating T_reg_, activated CD4^+^ T cells, IL-17-producing T_H_17 cells, and IL-6-producing monocytes (**Figure 1**) (96–101). While this may sound counterintuitive, a study reported a decreased capability of PD-isolated T_reg_ to suppress the activity of effector T cells *in vitro* (96, 97). In fact, the levels of blood cytokines found in PD patients correlate with an increase in sensory hypersensitivity. Thus, CD4^+^ T cells in patients experiencing pain have a lower IL-6/IL-10 ratio, while CD8^+^ T cells display a higher TNF-α/IL-10 ratio (102).

## Peripheral Mechanisms of PD-Induced Pain

Compared with ~5% of the general population, 20%–60% of PD patients show large- and small-fiber PN (73, 103). Interestingly, the severity of large-fiber neuropathy is also a marker of PD severity (104). Skin biopsies of PD patients with sensory hypersensitivity revealed that α-synuclein aggregates in cutaneous sensory nerves and leads to their degeneration (105–109). In a study of 72 PD patients, damage found in Aδ sensory fibers correlated with the level of sensory hypersensitivity they experienced. As a functional indication for abnormal pain fiber inputs, hypersensitive PD patients have a lower pain threshold to electrical stimuli as well as a higher current perception threshold than their normosensitive counterparts (110).

By contrast, Nolano et al. found that PD patients (n=18 subjects) were generally hyposensitive. They explained these findings as the loss of epidermal nerve fibers and Meissner corpuscles, which translated into an increase in tactile and thermal thresholds (105). A more recent study by the same authors analyzed skin innervation in 85 PD patients and found a significant reduction in intraepidermal nerve fiber density. This phenotype was correlated with a decrease in the perception of mechanical pain (107). However, further clinical studies are needed to clarify any putative correlation between PD pain thresholds, peripheral nerve degeneration, levels of dermal α-syn, and immunocyte infiltration.

Circulating α-syn-reactive CD4^+^ T cells are expanded in PD patients’ blood (**Figure 1**) (111). When injected into the gastrocnemius muscle of PD mice (M83 model), α-Syn preformed fibrils (PFF) aggregate in the dorsal nerve roots and lumbar DRG sensory neurons, as well as the lumbar spinal cord, the midbrain PAG matter, and the thalamus. When α-Syn aggregates in sensory neurons, it decreases nerve conduction velocity, drives small- and medium-sized myelinated fiber pathology, and induces mechanical allodynia (112). Importantly, these alterations were observed in the absence of motor dysfunction.

Injecting 1-methyl-4-phenyl-1,2,3,6-tetrahydropyridine (MPTP; i.p. 11 mg/kg, daily for five days), a pro-neurotoxin used to model PD, led to an increase in sensory neuron expression of Na_V_1.1, Na_V_1.7, Na_V_1.9. These alterations occurred 12 days after the first MPTP challenge and were accompanied by thermal hypersensitivity (confirmed by the hot plate and tail-flick tests) and extensive loss of striatal dopamine (113). In reserpine-injected rats presenting with mechanical hyperalgesia, neuron-profiling data showed increased expression of the acid-sensing ion channel ASIC3. Its specific blockade reversed reserpine-induced pain. This effect was accompanied by sustained spinal dorsal horn microglial activation, whose inhibition with minocycline reversed mechanical hyperalgesia (114).

Using a PD animal model, these studies demonstrate the presence of functional and transcription alterations in the somatosensory nervous system. They also provide a mechanistic link between the expression of prototypical pain-associated ion channels receptors and the onset of PD non-motor symptoms. Further research is necessary to test whether targeting these changes would, along with stopping PD-induced pain, alleviate CNS alteration by delaying or preventing the onset of PD motor symptoms.

## Central Mechanisms of PD-Induced Pain

In PD patients suffering from chronic pain, positron emission tomography (PET) studies showed increased neuronal activity in the prefrontal cortex, the primary somatosensory cortex, the posterior insula, and the anterior cingulate cortex (84, 115). At resting-state, magnetic resonance imaging (MRI) analysis of the connectivity between the right nucleus accumbens and the left hippocampus showed that it was reduced in PD patients experiencing pain compared with pain-free PD patients (116, 117). Before there is any noticeable degeneration in the substantia nigra and the onset of motor symptoms, early signs of PD neuropathology are first found in the locus coeruleus and raphe nuclei. Notably, these two supraspinal regions are typically associated with pain processing (118).

**Figure 2:**
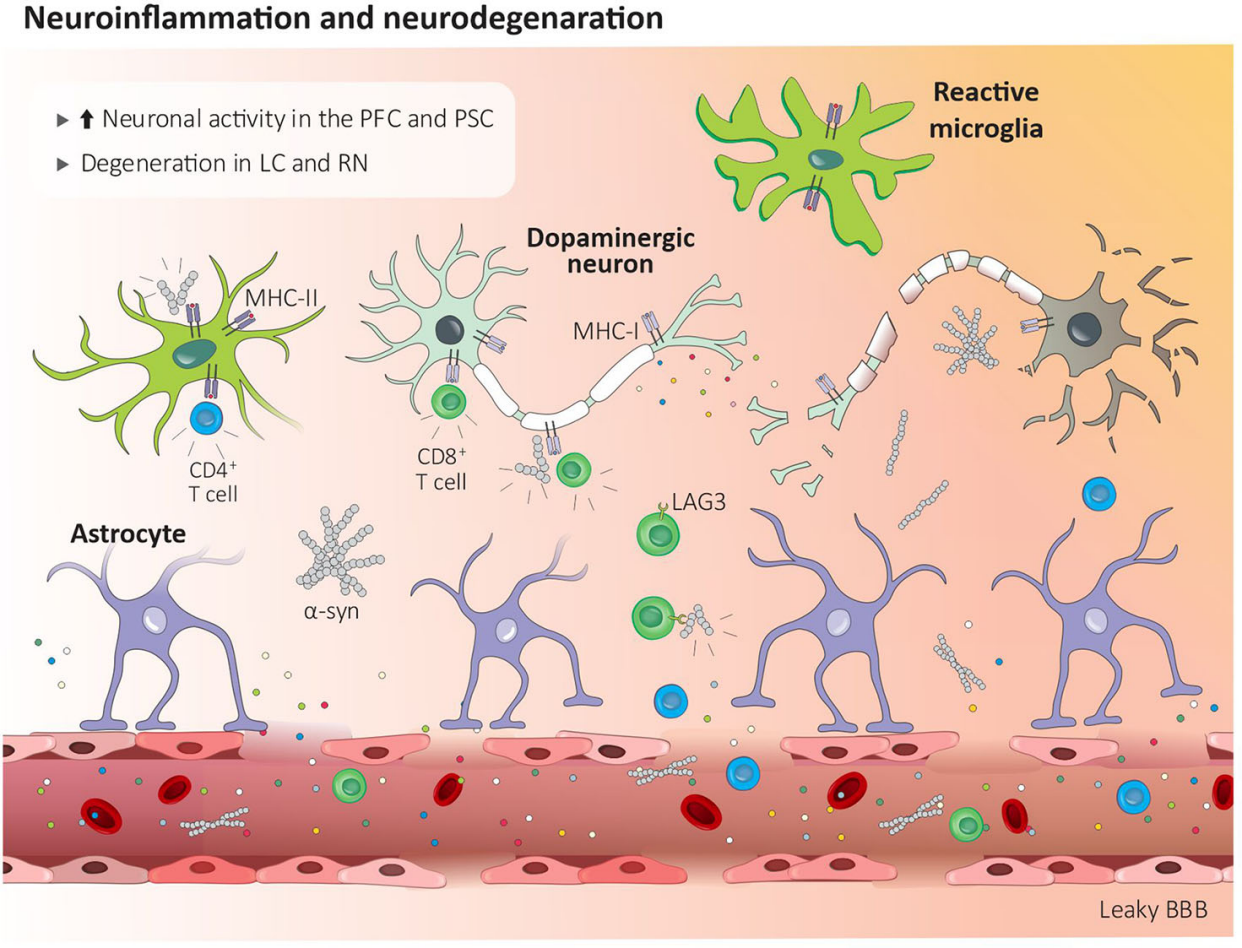
Central inflammation in Parkinson’s disease. Peripheral inflammation increases the blood brain barrier permeability which facilitates the infiltration of CD4^+^ and CD8^+^ T cells. Lag3^+^ CD8 T cells help disseminate α-syn centrally, while brain circulating α-syn-reactive CD4^+^ and CD8^+^ T cells recognize MHC-II bound peptides on microglia and MHC-I on dopaminergic neurons. Modified α-synuclein acts as a damage-associated molecular pattern, and *via* its action on receptors found on microglia and macrophages, induces cytokines release and neurodegeneration. BBB, blood brain barrier; LAG3, lymphocyte activation gene-3; LC, locus coeruleus; MHC-I, major histocompatibility complex I; MHC-II, major histocompatibility complex II; PFC, prefrontal cortex; PSC, primary somatosensory cortex; RN, raphe nuclei; α-syn, α-synuclein .

α-syn aggregates are present in the regions associated with pain processing including the lamina I of the spinal cord, the preganglionic neurons of the vagal nerve, and the sympathetic preganglionic neurons as well as in the coeliac ganglion (119). Blood-purified CD4^+^ and CD8^+^ T cells from PD patients recognize and respond to α-syn, which leads to the production of IL-5 or IFN-γ (**Figure 1**) (120). Brain circulating α-syn-specific CD4^+^ and CD8^+^ T cells can recognize specific peptides bound to MHC-II on microglia and MHC-I on dopaminergic neurons (111). In addition, effector CD8 T cells expressing the immune checkpoint receptors lymphocyte activation gene-3 (LAG3) bind with pathogenic α-syn, which favors its endocytosis and central dissemination (**Figure 2**) (121).

Along with these changes, peripheral inflammation also increases the permeability of the blood-brain barrier (BBB), which facilitates the infiltration of pathogenic lymphocytes in the CNS. CD4^+^ and CD8^+^ T cells were found in the brain parenchyma of PD patients as well as in different PD animal models. Although the mechanisms are not clearly understood, there is evidence indicating that infiltrated T cells drive neurodegeneration through the release and action of cytokines on their cognate receptors, which are found on CNS neurons (**Figure 2**). For instance, CD8 cytotoxic T cells’ TCR recognize MHCI-expressed mitochondrial antigens expressed by dopaminergic neurons and were posited to drive their elimination by a mechanism that is not fully understood (**Figure 2**) (122). Another example of T cell-mediated neurodegeneration stems from IL-17-derived from CD3^+^ T cells, which, when co-cultured, eliminate PD patients’ iPSC-derived midbrain dopaminergic neurons (98).

## GI Dysfunction in PD-Induced Pain

Gastrointestinal (GI) alterations are found in up to 80% of PD patients (123–125), with symptoms ranging from constipation to nausea, dyspepsia, and dysphagia (126). Constipation might precede the onset of PD motor symptoms by several decades (127, 128) and has been considered a prodromal hallmark of PD (126). In PD patients, severe GI symptoms are predictive of impaired cognitive performance.

**Figure 3:**
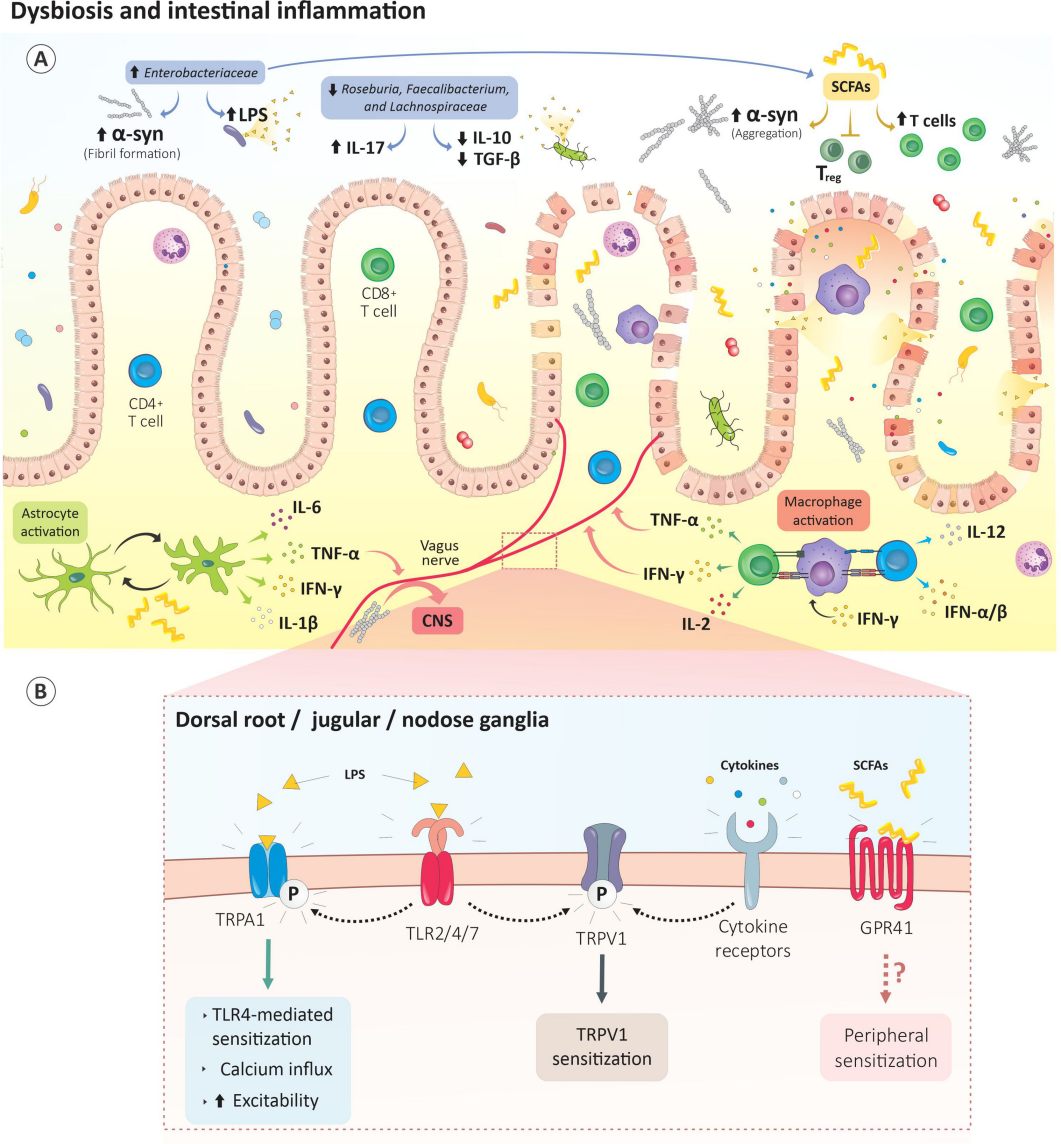
Gut dysbiosis drives PD-induced pain. **(A)** The gut dysbiosis in PD is characterized by enhances *Enterobacteriaceae* content. These bacteria increase LPS levels which subsequently promote gut permeability. Raises in *Enterobacteriaceae* metabolites, such as short-chain fatty acids, promote α‐syn fibril formation, microglia activation and blunt T_reg_-mediated immunosuppression. Proteolyzed α-syn by neurons and antigen presenting cells is then presented by the MHC machinery and leads to activation of autoreactive CD8 and CD4 T cells. **(B)** The dysbiotic bacteria and their metabolites directly activate vagal or ENS neurons. For instance, LPS activates nociceptor neurons-expressed TLR2/4/7 while SCFAs sensitize GPR41-expressing neurons. Bacteria-activated or α-syn-autoreactive immunocytes released cytokines can also activate their cognate receptors on sensory neurons. Subsequently, intracellular mechanisms are triggered in these neurons and culminate in TRP channels phosphorylation, neurons sensitization and sensory hypersensitivity. CNS, central nervous system; GPR41, G protein-coupled receptor 41; IFN-γ, interferon γ; IL-1β, interleukin 1 beta; IL-2, interleukin 2; IL-6, interleukin 6; IL-10, interleukin 10; IL-12, interleukin 12; IL-17, interleukin 17; LPS, lipopolysaccharide; TGFβ, transforming growth factor beta; TLR2/4/7, Toll-like receptor 2/4/7; T_reg_, regulatory T cells TRPA1, transient receptor potential ankyrin-like 1; TRPV1, transient receptor potential cation channel subfamily V; TNF-α, tumor necrosis factor; SCFAs, short-chain fatty acids; α-syn, α-synuclein .

α-syn, which is typically found in CNS of PD patients, is also present in the colon, the neurons of the ENS, and the vagus nerve (129, 130). These findings were phenocopy in animal models of PD, in which aggregates of α-syn were found in the GI tract (**Figure 3A**) (131–134). As such, a growing number of investigators argue that PD might start in the gut and spread to the CNS through the vagus nerve (**Figure 3A**). This hypothesis is supported by animal studies showing that exogenous α-syn injected into the gut wall migrated to the brain *via* the vagus nerve at a rate estimated to be 5–10 mm/day in rats (135), while a patient who underwent a truncal vagotomy showed a decreased risk of developing PD later in life (136, 137). Although the cause of these disruptions and their role in PD pathogenesis remains unclear, the presence of α-syn in the ENS is sufficient to induce colonic dysmotility in the Gl tract, which correlates with the severity of motor impairment in some animal models (138, 139).

PD patients experiencing constipation showed increased infiltration of CD4^+^ T cells into the colonic mucosa as well as elevated circulating T_H_17 and T_reg_ cells (140). In addition, PINK1 or Parkin deficient mice exposed to bacterial intestinal infection showed an increase in BBB permeability, which facilitates the influx of cytotoxic CD8 T cells into the CNS. These pro-inflammatory cytotoxic CD8 T cells target the host’s mitochondrial antigens. Such auto-immune reactions can lead to the elimination of dopaminergic neurons in the striatum and subsequent motor impairments (141). Finally, histologic data showed increases in the immunoreactivity of the astrocytic marker GFAP in the colon of PD patients as well as increases in TNF-α, IFN-γ, IL-6, and IL-1β levels (142) (**Figure 3A**). These mediators were elevated in the early stages of the disease and were negatively correlated with disease duration (142). Given that enteric and central glial cells respond to IL-6, and IL-1β, and that their upregulation is associated with inflammatory pain (143), it is conceivable that the influx of cytotoxic CD8 T cells heightens pain transmission centrally or within the gut wall.

## Dysbiosis in PD-Induced Pain

In conjunction with gastrointestinal dysfunction and inflammation, gut dysbiosis may contribute to PD progression by increasing the permeability of the blood-gut barrier and BBB and facilitating the transport of peripheral α-syn to the brain (138). Similarly, microbes can directly activate sensory neurons to trigger pain hypersensitivity (see *Immunity in PD*). As such, PD-induced dysbiotic bacteria and their metabolites may activate vagal or ENS neurons directly, or indirectly *via* activated immunocyte-released cytokines (**Figure 3B**) (21, 144).

Such dysbiosis is characterized by increased levels of *Enterobacteriaceae, Akkermansia* spp.*, Catabacter* spp., and *Akkermansiaceae* and a decreased level of *Roseburia* spp.*, Faecalibacterium* spp., and Lachnospiraceae (145, 146). While the function of these bacteria is diverse and is likely to be context-dependent, some patterns are evident. *Roseburia* spp. and *Faecalibacterium* spp. are typically known for their anti-inflammatory properties. *Faecalibacterium* spp. would blunt CD4 differentiation to T_H_17 cells and promote differentiation to T_reg_ (147–149). *Roseburia* spp. and *Faecalibacterium* spp. would also downregulate IL-17 expression (150), and promote the release of the anti-inflammatory cytokines IL-10 and TGF-β (**Figure 3A**) (151, 152).

*Roseburia* spp. was found in increased levels in the blood and stool samples of fibromyalgia patients (153) and in stool samples of obese patients with back pain (154). When supplemented, *Roseburia* spp. alleviates stress-related visceral pain (155). It is therefore conceivable that reduced levels of *Roseburia* spp. and *Faecalibacterium* spp. exacerbates T_H_17 activity and/or limits the T_reg_ which promotes PD-induced pain.

In the stools of PD patients, Enterobacteriaceae levels correlate with motor symptoms (146) and are associated with increased lipopolysaccharide (LPS) levels and α‐syn fibril formation (**Figure 3A**) (156). While no direct link exists between Enterobacteriaceae and PD-induced pain, LPS is known to activate nociceptor neurons expressed by TLR2, -4 and -7 (157) and, possibly, TRPA1, TRPM3, TRPM8, and TRPV1 (158, 159). Enterobacteriaceae-mediated increases in LPS levels may therefore lead to the sensitization of gut-innervating nociceptor neurons (**Figure 3B**).

Bacteria produce various short-chain fatty acid (SCFA) metabolites including acetate, propionate, butyrate, and valeric acid. In the gut of SPF mice that underwent a CCI, these SCFAs drive hippocampal microglia polarization and IL-1β and TNF-α release. These cytokines, in turn, mediate mechanical and thermal hyperalgesia (160). When administered to germ-free α-syn overexpressing mice, SCFAs increased α-syn aggregation and microglia activation and contributed to motor dysfunction (161). SCFAs were also shown to modulate microglia activation during viral infections (162) (**Figure 3A**). Therefore, SFCA-mediated microglial activation may drive the central sensitization of pain pathways.

SCFAs blunt T_reg_-mediated immunosuppression and increase T cell density (163–165) (**Figure 3A**). By activating GPR43 and GPR41, SCFA inhibits the leukocytes’ histone deacetylase (HDAC) which, in turn, increase the leukocyte chemotaxis, chemokine production, and the expression of adhesion molecules (166). Given that sodium butyrate, an HDAC inhibitor diminishes CCI-induced TNF-α release and pain (167), it can be surmised that PD-induced gut SCFAs can raise circulating cytokine levels (i.e., TNF-α) and promote sensory hypersensitivities by increasing the immunomodulatory action of HDAC in leukocytes Finally, SCFA were found to sensitize GPR41-expressing lumbar and vagal neurons (**Figure 3B**) (168).

Fecal microbiota transplantation (FMT) was found to rescue gut dysbiosis, decrease SCFAs levels, alleviate physical impairment, and increase striatal DA and 5-HT content in PD animal models. Microglia and astrocyte activation was diminished in the substantia nigra, and neuroinflammation was suppressed by reducing TLR4/TNF-α signaling (169). In PD patients, FMT reduced constipation and, albeit transiently, leg tremors (170, 171). A preliminary study with 15 PD patients reported that colonic FMT administration alleviates motor and non-motor symptoms, while improving anxiety, depression, and sleep quality scores (172). In another prospective study, 11 PD patients who underwent FMT had reduced constipation and improved postural instability and gait (173).

Modulating pain by rescuing a healthy microbiome has also been postulated (174). Preclinical and clinical studies indicate that probiotic consumption alleviates visceral pain (175). Based on these findings, along with the fact that PD is characterized by dysbiosis, and that bacteria and their metabolites modulate sensory neuron function, we hypothesize that FMT may improve PD-induced pain and delay the onset of motor symptoms.

## Conclusion

**Figure 4:**
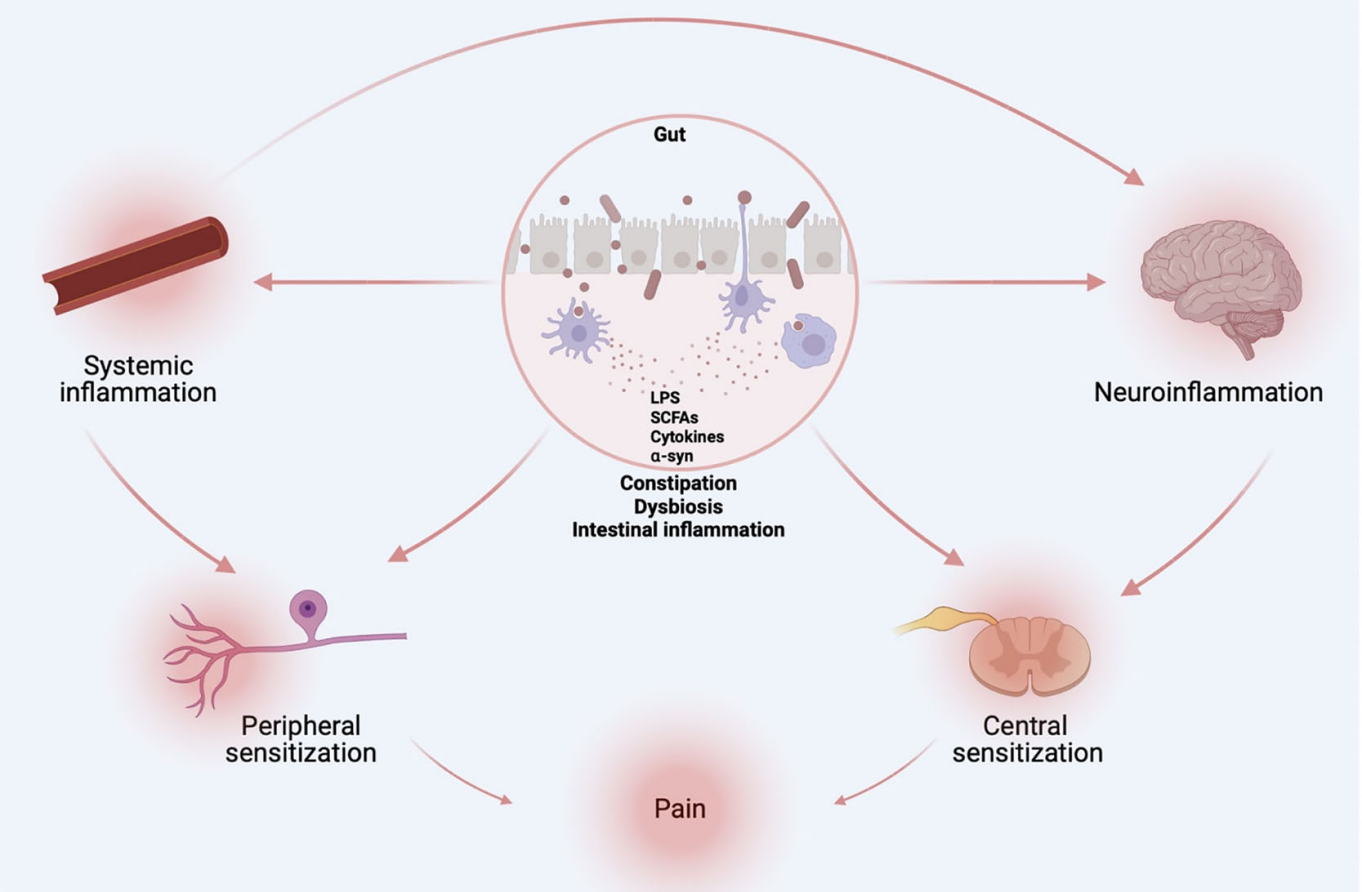
Origins of PD-induced pain. Gut dysbiosis, altered peripheral and central immunity, and increased local (i.e., gut) and systemic cytokine content, as well as increased gut-brain barrier and BBB permeability are likely drivers of sensory hypersensitivity in PD. Figure created with biorender.com.

Along with constipation, sensory hypersensitivity precedes the onset of motor symptoms in PD. These physiological alterations are accompanied by gut dysbiosis, altered peripheral and central immunity, and increased local (i.e., gut) and systemic cytokine content, as well as increased gut-brain barrier and BBB permeability. We posit that gut dysbiosis leads to systemic inflammation, which drives sensory hypersensitivity (**Figure 4**). *Via* local pro-inflammatory loops, these hypersensitized nociceptor neurons are likely to amplify immune responses and speed up central neurodegeneration. Alleviating constipation, rescuing microbiota *via* fecal matter transplant, blocking leukocyte activity and cytokine action on neurons using targeted antibodies, and limiting BBB and gut hyperpermeability all constitute potential ways of preventing neuro-immune and microbe-neuron interplays and subsequent pain hypersensitivity. Further studies should investigate how nociceptor neurons increase activity, would, in turn, modulate dysbiosis and central neurodegeneration. Should pain constitute an early driver of PD pathophysiology, monitoring and alleviating this symptom may constitute a novel biomarker and therapeutic target to slow the progression of PD.

## Author Contributions

KR and ST designed the study. KR, NC-S, KrR, MG, CB, RP, and ST wrote the manuscript. All authors contributed to the article and approved the submitted version.

## Funding

ST is financed by Canada Research Chair program (#950-231859), Canadian Institutes of Health Research (#162211, #461274, #461275), the Canadian Foundation for Innovation (#37439), and Natural Sciences and Engineering Research Council of Canada (#RGPIN-2019- 06824), as well as the Fonds de recherche du Québec – Santé and Centre interdisciplinaire de recherche sur le cerveau et l'apprentissage (CIRCA). KR holds postdoctoral fellowships from the Fonds de recherche du Québec - Nature et technologies (FRQNT; #289949), CIRCA and Fonds de recherche en ophtalmologie - Université de Montréal (FROUM). KrR holds postdoctoral fellowships from CIRCA and FROUM. NCS holds a Merit scholarship from the Faculty of Medicine of the Université de Montréal.

## Conflict of Interest

ST has an equity stake in Nocion Therapeutics.

The remaining authors declare that the research was conducted in the absence of any commercial or financial relationships that could be construed as a potential conflict of interest.

## Publisher’s Note

All claims expressed in this article are solely those of the authors and do not necessarily represent those of their affiliated organizations, or those of the publisher, the editors and the reviewers. Any product that may be evaluated in this article, or claim that may be made by its manufacturer, is not guaranteed or endorsed by the publisher.
